# Use of Sotrovimab in a Pregnant Patient With COVID-19 Infection

**DOI:** 10.7759/cureus.22658

**Published:** 2022-02-27

**Authors:** Isha Gupta, Esther S Arguello Perez

**Affiliations:** 1 Nephrology, Middletown Medical, Middletown, USA; 2 Nephrology, Garnet Health Medical Center, Middletown, USA; 3 Internal Medicine/Nephrology, Touro College of Osteopathic Medicine, Middletown, USA; 4 Infectious Disease, BronxCare Health System, New York, USA

**Keywords:** monoclonal antibodies, monoclonal antibody, sars-cov-2, omicron, pregnancy, coronavirus 2019, sotrovimab, covid-19, pregnant

## Abstract

For individuals with mild-to-moderate coronavirus disease 2019 (COVID-19, caused by severe acute respiratory syndrome coronavirus 2 [SARS-CoV-2]), monoclonal antibodies (MOABs) are known to prevent progression of the disease and hospitalization. Pregnant women, who are at an increased risk of severe COVID-19 infection, have been significantly underrepresented in studies for MOAB treatments, especially sotrovimab. Specifically, there has only been one case reported of a pregnant woman using sotrovimab successfully. We report a second such patient - an unvaccinated 21-year-old, COVID-19-positive, 16-week pregnant woman who was followed closely over the next 60 days post-MOAB infusion. We noted prevention in the progression of the disease and hospitalization without any fetal/pregnancy-related complications.

## Introduction

The Centers for Disease Control and Prevention (CDC) has reported 173,508 cases and 274 deaths of pregnant women due to coronavirus disease 2019 (COVID-19) in the United States between January 22nd 2020 to February 14th 2022 [1]. Pregnancy is a known risk factor for severe illness from COVID-19 infection [2]. The risk of ICU admission, requirement of invasive mechanical ventilation and death has been reported significantly higher amongst pregnant people compared to non-pregnant people [3]. There is also an increased risk of adverse obstetric outcomes amongst pregnant people with COVID-19, such as pre-term or stillbirth [4,5].

Within the general patient population, the activity of monoclonal antibodies (MOABs) against the spike protein of COVID-19 has been reported to decrease the risk of hospitalization and death [6-8]. On November 9th 2020, Emergency Use Authorization (EUA) was first issued to use MOABs in COVID-19 patients at risk of progression of the mild-to-moderate disease to a more severe form [9]. On May 14th 2021, the FDA included pregnancy as a qualifying condition for the use of these antibodies. With the rise of Omicron variant in December 2021, sotrovimab became the only MOAB effective against COVID-19 infection in non-hospitalized patients at the time [10]. Upon review of data in PubMed until February 20th 2022, only one case report about sotrovimab use in a COVID-19 pregnant patient has been described [11]. The case below outlines the second such case.

## Case presentation

The patient is an unvaccinated 21-year-old, 16-week pregnant woman, with a medical history of mild intermittent asthma. She first noticed symptoms on November 30th 2021, while she officially tested positive on December 6th (Table 1). During the follow-up telemedicine visit on December 7th, the patient revealed that while she was not experiencing fever, shortness of breath or a drop in oxygen saturation level below 95% (self-monitored), she had been dealing with cough, nausea, sore throat, decreased sense of smell, chest congestion and headaches for the past week.

**Table 1:** COVID-19 testing SARS-CoV-2: severe acute respiratory syndrome coronavirus 2, RT-PCR: reverse transcription polymerase chain reaction, CT: cycle threshold

TEST	RESULT	REFERENCE RANGE
SARS-CoV-2 RT-PCR	Positive	Negative
ORF1a gene	20.2 (CT Values)	(CT Values) Not detected< 10 or > 38 Detected 10 .0 – 38.0
N gene	20.6 (CT Values)	(CT Values) Not detected< 10 or > 38 Detected 10 .0 – 38.0

Given the risk factors of pregnancy, her vaccination status and the presence of mild symptoms of COVID-19, the patient was offered MOAB infusion. After consulting with her obstetrician, the patient accepted the infusion treatment. On December 8th 2021, the patient received sotrovimab infusion (500 mg) mixed with normal saline over a 30-minute period. Her vitals were monitored through the infusion process and for another hour after it (Table 2). She tolerated it without any side effects.

**Table 2:** Vital Signs monitored during the monoclonal antibody (MOAB) treatment

Stage of treatment	Temp (F)	BP (mmHg)	Pulse	RR	Oxygen sat. (%)
Pre-infusion	98.7	144/68	73	20	100
During-infusion	98.9	134/69	75	24	100
Post-infusion	99.6	131/72	94	25	99

The patient had a telemedicine follow-up the next day, when she reported a significant improvement in her cough, nausea, headaches, and fatigue and felt only mild chest congestion at that time. She was also followed up on Day 14, Day 30, and Day 60 post infusion. The patient reported resolution of her symptoms and confirmed that she did not need any urgent care or emergency room visits or hospitalization during the follow-up period. No fetal or pregnancy-related complications were noted by her obstetrician, during her two monthly follow-ups since the infusion. 

## Discussion

On March 11th 2020, the World Health Organization (WHO) declared COVID-19 to be a global pandemic, its first such designation since declaring H1N1 influenza a pandemic in 2009. The pandemic has claimed about 5.8 million lives worldwide, out of which 921,984 deaths have been reported in the United States alone, as per the WHO database on February 18th, 2022. In the initial phase of the pandemic, different drugs and therapies like ivermectin, convalescent plasma, hydroxychloroquine/chloroquine, nitazoxanide etc. were used to treat COVID-19 infection, but their use was soon dissuaded by COVID-19 Treatment Guidelines Panel. Development of MOABs marked a major breakthrough in management of SARS-CoV-2, leading to Emergency Use Authorization (EUA) on November 9th 2020 in COVID-19 patients at risk of progression of the mild-to-moderate disease to a more severe form [9]. Six months later, the FDA included pregnancy as a qualifying condition for the use of MOABs, supported by the National Institutes of Health, though there continues to be very limited data looking at the long-term efficacy and potential risk of poor maternal/fetal outcomes [12].

SARS-CoV-2 variant B.1.1.529 (named Omicron) was first reported to the WHO on November 24th 2021 and was designated a variant of concern (VOC) on November 26th 2021. Since December 26th 2021, the Omicron variant has accounted for more than 90% of the COVID-19 cases in the USA [10]. Until then, there were three MOABs that were widely available for outpatient management of coronavirus patients. On January 24th 2022, the FDA updated EUA factsheets to discontinue the use of the other two, leaving sotrovimab as the only approved MOAB to be used on COVID-19 positive outpatient management [13-15].

Managing COVID-19 in pregnant patients has imposed a big challenge, especially due to absence of large-scale clinical trials. Previously, only a few retrospective case reports and case series have been published with experience in using banlanivimab/etesevimab and casirivimab/imdevimab in pregnant women [16-20]. They have generally shown favorable outcomes without significant side effects. Only Chang and Richley reported adverse reactions during infusion of MOAB, in one and two patients respectively [19,20].

Sotrovimab is a pan-sarbecovirus monoclonal antibody. Its efficacy against COVID-19 in high risk patients has been studied in COMET-ICE (a Phase III, multi-center, double-blinded, placebo-control trial) and proven to reduce risk of progression of mild to moderate infection to severe form. The study, however excluded any women that were pregnant, breast-feeding or had childbearing potential (lack of effective contraception). To this day (February 20, 2022), safe and successful use of sotrovimab has only been reported in one case report in a pregnant patient (with kidney transplant) [11].

## Conclusions

Our patient was pregnant and unvaccinated against COVID-19, which are well-known risk factors for progression of mild to moderate coronavirus to severe form and for need for hospitalization. The outcome of the sotrovimab infusion in our patient was favorable, with rapid resolution of symptoms, prevention of hospitalization and prevention of progression to severe infection. However, there continues to be a dire need for larger-scale studies to derive definitive short and long-term benefits in this patient population.
